# Whole-genome sequencing-based characterization of resistome and virulome in *Klebsiella pneumoniae* isolated from ready-to-eat foods

**DOI:** 10.3389/fmicb.2026.1788677

**Published:** 2026-04-15

**Authors:** Nuha Anajirih, Sulaiman A. Anagreyyah, Hanka Brangsch, Shawky Moustafa, Dalia F. Khater, Ahmed S. Gesraha, Amira M. Rizk, Heinrich Neubauer, Gamal Wareth

**Affiliations:** 1 Department of Medical Emergency Services, Faculty of Health Sciences in Al-Qunfudah, Umm Al-Qura University, Makkah, Saudi Arabia; 2 Department of Family Medicine, King Fahad Armed Hospital, Jeddah, Saudi Arabia; 3 Institute of Bacterial Infections and Zoonoses, Friedrich-Loeffler-Institut, Jena, Germany; 4 Department of Pathology, Faculty of Veterinary Medicine, Benha University, Toukh, Egypt; 5 Tanta Laboratory, Animal Health Research Institute, Agricultural Research Center, Tanta, Egypt; 6 Department of Pharmacology, Medical Research and Clinical Studies Institute, National Research Centre, Giza, Egypt; 7 Department of Bacteriology, Immunology, and Mycology, Faculty of Veterinary Medicine, Benha University, Toukh, Egypt; 8 Institute of Infectious Diseases and Infection Control, Jena University Hospital, Jena, Germany

**Keywords:** *Klebsiella pneumoniae*, ready-to-eat food, resistomes, virulomes, WGS

## Abstract

*Klebsiella* (*K.*) *pneumoniae* is a major antimicrobial-resistant pathogen of global concern. It has increasingly been reported outside clinical settings, including food products. However, genomic data on food-derived *K. pneumoniae* in Egypt remain limited. In this study, we investigated the genomic diversity, antimicrobial susceptibility, and phylogenetic relationships of *K. pneumoniae* isolated from ready-for-consumption foods obtained from Egyptian supermarkets. Eleven isolates were recovered from dairy products (milk and yogurt) and catfish. Isolates were identified by MALDI-TOF MS and confirmed by whole genome sequencing (WGS). Antimicrobial susceptibility testing (AST) was performed against a panel of clinically relevant antibiotics. Genomic analyses included multilocus sequence typing (MLST), detection of resistance and virulence associated genes, and SNP based phylogenetic reconstruction. The SNP-based phylogenetic analysis was performed using an additional 77 publicly available Egyptian clinical isolates. Most isolates displayed a predominantly susceptible antimicrobial phenotype. Resistance was largely restricted to piperacillin. All genomes carried intrinsic resistance determinants, including multiple *bla*SHV alleles and *fos*A variants. These did not correlate with phenotypic resistance to cephalosporins or fosfomycin. MLST revealed heterogeneous lineages, including clinically relevant sequence types ST37 and ST105, as well as a novel sequence type. Phylogenetic analysis showed that some food-derived isolates clustered closely with the genomes of Egyptian clinical isolates. Others formed genetically distinct lineages, indicating diverse origins within the food chain. These findings show that ready-to-eat foods in Egypt can harbor genetically diverse *K. pneumoniae* strains. Some populations include lineages related to clinical strains, despite limited phenotypic resistance. The study highlights the importance of integrating phenotypic antimicrobial testing with WGS based surveillance. Such integration can better assess the public health significance of foodborne *K. pneumoniae* within a One Health framework.

## 1 Introduction

*Klebsiella* (*K.*) *pneumoniae* is an opportunistic Gram-negative bacterium of the *Enterobacteriaceae* family, widely recognized as a significant cause of community- and healthcare-associated infections, including pneumonia, bloodstream infections, urinary tract infections, and liver abscesses (Martin and Bachman, 2018; Asokan et al., 2025). In recent decades, the global epidemiology of *K. pneumoniae* has been shaped by the emergence and dissemination of multidrug-resistant (MDR) and hypervirulent *K. pneumoniae* (hvKp) strains, often carrying extended-spectrum β-lactamases (ESBLs), carbapenemases, and other clinically relevant resistance determinants (Wyres and Holt, 2018; Pitout et al., 2015; Alhaji et al., 2025). These traits, combined with the organism’s ability to acquire mobile genetic elements (MGEs) and form biofilms, complicate treatment and facilitate persistence in diverse environments (Paczosa and Mecsas, 2016). *K. pneumoniae* is a significant environmental pathogen found in soil, water, and on plants. It survives well outside the body, particularly in moist environments such as sinks, medical equipment, and wastewater, which facilitates its spread. Environmental *K. pneumoniae* acquires resistance genes via MGEs from other microorganisms in environmental sources such as polluted soil and water, enters humans through contaminated food and water, and then transfers these genes to the body and healthcare settings (Araújo et al., 2025). Although *K. pneumoniae* is primarily associated with human infections, accumulating evidence demonstrates its occurrence in food products of animal and plant origin, suggesting a potential role of the food chain as a reservoir and transmission route (Klaper et al., 2021; Zhang et al., 2018). Retail meats, seafood, dairy products, fresh produced, and ready-to-eat (RTE) foods have all been reported as sources of *K. pneumoniae*, some carrying high-risk sequence types (STs), antimicrobial resistance (AMR) determinants, and hypervirulent capsular types such as K1, K2, and K54 (Rodrigues et al., 2022; Bogomazova et al., 2020). A multicentric European survey, for example, found *K. pneumoniae* in 60% of chicken meat and 30% of RTE salads, with identical genotypes across products, underscoring the role of food as a vehicle for dissemination (Rodrigues et al., 2022). Complementary whole genome sequencing (WGS) studies of isolates from animals and food in Germany revealed wide genetic diversity, frequent ESBL genes, and virulence loci such as *ybt* and *iuc*, further illustrating the One Health relevance of food and animal reservoirs (Klaper et al., 2021; Wareth et al., 2022).

In Egypt, *K. pneumoniae* has been detected in several food sources, including RTE luncheon meats, where 12.6% of processed meat samples were positive, and isolates frequently carried ESBL genes (*bla*SHV, *bla*TEM, and *bla*CTX-M-15), *ampc*, and carbapenemase determinants, alongside hypermucoviscous K1 and K2 serotypes (Abdel-Rhman, 2020). Other Egyptian studies reported *K. pneumoniae* in poultry and farm environments (Elmonir et al., 2021), bivalves (Mohammed et al., 2024), and fresh products from farms, where isolates from irrigation water (30%) and tomatoes carried carbapenemase (*bla*NDM and *bla*OXA-48) and ESBL genes (*bla*SHV and *bla*TEM) (Elshafiee et al., 2022). Notably, colistin resistance and hypervirulence genes have also been detected, raising concerns about limited therapeutic options and increased pathogenic potential (Elmonir et al., 2021; Mohammed et al., 2024). Despite these findings, comprehensive genomic investigations of *K. pneumoniae* from RTE foods in Egypt remain limited, and the genetic relatedness of these isolates to clinical strains has not been fully elucidated.

This study aimed to investigate the antimicrobial resistance profiles and genomic characteristics of *K. pneumoniae* isolated from various RTE food products obtained from Egyptian supermarkets. Whole genome sequencing (WGS) was performed to comprehensively characterize AMR determinants, virulence associated genes, and sequence types. By integrating phenotypic and genomic data, this work provides novel insights into the potential public health risks posed by foodborne *K. pneumoniae* in Egypt. It contributes to the global One Health understanding of its epidemiology within the food supply chain.

## 2 Materials and methods

### 2.1 Bacterial isolates and identification

A total of 11 *Klebsiella* spp. isolates were obtained from analyzing 178 food products intended for human consumption, collected from supermarkets in 2017 in Egypt. The isolates were recovered from milk (*n* = 5), catfish (*n* = 5), and yogurt (*n* = 1). The milk samples were obtained from Kutour City, the catfish samples from Tanta, and the yogurt sample from Kafr El Zayat, all located within the Delta Region of Egypt. *K. pneumoniae* strains were transferred to the Institute of Bacterial Infections and Zoonoses (IBIZ, Jena, Germany) for confirmation and molecular typing. Nagoya approval for transferring the samples has been obtained. Metadata for all samples are presented in Supplementary Table 1. Species-level identity of all isolates was verified using Matrix-Assisted Laser Desorption/Ionization Time-of-Flight Mass Spectrometry (MALDI-TOF MS) with a log score value >2.300. Protein extraction from pure colonies was performed following previously described protocols (Khater et al., 2021). MALDI-TOF analyses were performed with a Microflex LT instrument (Bruker Daltonics, Bremen, Germany) according to the MALDI Biotyper manufacturer’s guidelines for species identification (log score range 0–3). Scores between 2.300 and 3.000 were interpreted as “highly probable species identification,” scores of 2.000–2.290 as “secure genus identification,” scores of 1.700–1.990 as “probable genus identification,” and scores below 1.690 were regarded as unreliable for identification. To further confirm the genus and species identity of each strain, whole genome sequencing data were analyzed using Kraken (v2.0.7_beta) (Wood et al., 2019) in combination with the MiniKraken v2 database to classify sequencing reads and assemblies and to assess potential contamination. For genus and species level identification, the top hit (highest percentage assignment) was considered.

### 2.2 Antibiotic susceptibility testing (AST)

Antibiotic susceptibility testing was performed by determining the Minimum Inhibitory Concentrations (MICs) through the broth microdilution technique. An automated MICRONAUT-S platform (MERLIN Diagnostics GmbH, Bornheim-Hersel, Germany) equipped with MDR MRGN-Screening plates was used for this purpose. The procedure followed established and previously published protocols (Wareth et al., 2020). The MICRONAUT-S software automatically categorized the strains as susceptible, intermediate (susceptible in increased exposure), or resistant based on MIC values, applying the breakpoint criteria defined by the Clinical and Laboratory Standards Institute for *K. pneumoniae*. Isolate susceptibility was assessed using a panel of 18 antimicrobial agents tested at different concentrations. The antibiotics set included cefepime (CEP), tigecycline (TGC), piperacillin/tazobactam (PIT), colistin (COL), ceftazidime/avibactam (CAA), amikacin (AMK), meropenem (MER), trimethoprim/sulfamethoxazole (T/S), ceftolozane/tazobactam (CTA), fosfomycin (FOS), chloramphenicol (CMP), ciprofloxacin (CIP), ertapenem (ERT), piperacillin (PIP), levofloxacin (LEV), cefotaxime (CTX), imipenem (IMP), and ceftazidime (CAZ).

### 2.3 WGS and *in silico* detection of AMR determinants and virulome

Genomic DNA was extracted using the High Pure PCR Template Preparation Kit (Roche Diagnostics GmbH, Mannheim, Germany) in accordance with the manufacturer’s guidelines. Library preparation was performed using the Nextera XT DNA Library Prep Kit (Illumina, San Diego, CA, USA), and sequencing was performed on the Illumina MiSeq platform using paired-end reads. Processing of the raw sequencing data was conducted with the Linux based pipeline WGSBAC (v2.0)^1^, as described previously (Linde et al., 2020). This pipeline includes quality assessment of the raw data using FastQC (v0.11.7) (Andrews, 2010). The pipeline also determines sequencing coverage of the raw data. Genome assemblies were generated using Shovill (v1.0.4)^2^, which employs the SPAdes assembler (Bankevich et al., 2012). Assembly quality was subsequently evaluated with QUAST (v5.0.2) (Gurevich et al., 2013). To screen for potential contamination, the sequence classifier Kraken 2 (v1.1) (Wood et al., 2019) in combination with the Kraken2DB database was applied. Prediction of virulence-associated genes was performed using ABRicate (v0.8.10)^3^ in conjunction with the Virulence Factor Database (Chen et al., 2005). To investigate genetic determinants associated with antimicrobial resistance, ABRicate was employed in combination with the Comprehensive Antibiotic Resistance Database (CARD) (Jia et al., 2017) and ResFinder (Zankari et al., 2012). Furthermore, AMRFinderPlus (NCBI) (Feldgarden et al., 2019) was utilized with parameters optimized for *Klebsiella* to detect resistance-related point mutations. Multilocus sequence typing (MLST) was performed *in silico* through the WGSBAC pipeline on assembled genomes, employing the mlst software (v2.16.1)^4^ with the *K. pneumoniae*–specific typing scheme. For core genome single nucleotide polymorphism (cgSNP) typing, Snippy v4.6.0^5^ was used with *Klebsiella pneumoniae* subsp. *pneumoniae* strain HS11286 (GCF_000240185.1) as reference. The genomes of the current set of isolates have been compared with 77 foreign Egyptian clinical isolates obtained from humans and animals in different localities in Egypt (Supplementary Table 2). Raw read data of these foreign isolates were downloaded from NCBI Short Read Archive. The cgSNP alignment was analyzed by Maximum likelihood analysis using RAxML v8.2.12 (Stamatakis, 2014) and the resulting tree was visualized using Microreact (Argimón et al., 2016).

## 3 Results

### 3.1 *Klebsiella* isolate identification and AST

All isolates were identified as *K. pneumoniae* using MALDI-TOF MS, and this identification was subsequently confirmed by whole genome sequencing. Taxonomic classification with Kraken2 further supported these findings, assigning all isolates to the genus *Klebsiella* with proportions ranging from 91.2% to 97.6%. At the species level, all isolates were assigned to *K. pneumoniae* (Supplementary Table 3). All 11 *K. pneumoniae* isolates were tested against a panel of 18 antibiotics. All isolates were susceptible to cefepime, tigecycline, piperacillin/tazobactam, colistin, ceftazidime/avibactam, amikacin, meropenem, trimethoprim/sulfamethoxazole, ceftolozane/tazobactam, fosfomycin, ciprofloxacin, ertapenem, levofloxacin, imipenem, and ceftazidime. However, reduced susceptibility was observed for a few antibiotics. For instance, three isolates (one from yogurt and two from milk) displayed reduced susceptibility (susceptible in increased exposure) to chloramphenicol. One isolate from catfish (Eg-BVM2) was resistant to cefotaxime and two isolates from catfish (Eg-BVM3, Eg-BVM5) and 4 from milk (Eg-BVM7, Eg-BVM8, Eg-BVM9, Eg-BVM11) showed resistant to piperacillin (Supplementary Table 1).

### 3.2 WGS analysis and assembly statistics

Whole genome sequencing of the 11 *K. pneumoniae* isolates yielded *de novo* assemblies of 34 contigs (Eg-BVM8) to 116 contigs (Eg-BVM10). N50 values ranged from 217,146 bp (Eg-BVM10) to 481,880 bp (Eg-BVM6). The total assembly sizes were consistent, between 5.4 and 5.7 Mb, with a GC content of 56.6%–57.3%, aligning with the expected genomic features of *K. pneumoniae* (Table 1).

**TABLE 1:** Metadata and genome assembly metrics of *Klebsiella pneumoniae* isolates (*n* = 11) from food samples in Egypt.

Metadata				Genome assembly metrics					
Isolate	ID	Food	Species	Contigs	N50 (bp)	GC (%)	Coverage (×)	Genome (Mb)	MLST ST
Eg-BVM1	17Y0567	Yogurt	*K. pneumoniae*	58	354,128	57.23	116	5.40	515
Eg-BVM2	17Y0667	Cat fish	*K. pneumoniae*	36	419,382	56.69	83	5.53	105
Eg-BVM3	17Y0668	Cat fish	*K. pneumoniae*	38	417,092	56.69	89	5.53	105
Eg-BVM4	17Y0669	Cat fish	*K. pneumoniae*	49	349,010	57.10	85	5.44	37
Eg-BVM5	17Y0672	Cat fish	*K. pneumoniae*	38	417,092	56.69	78	5.53	105
Eg-BVM6	17Y0673	Cat fish	*K. pneumoniae*	54	481,880	56.84	78	5.56	3063
Eg-BVM7	17Y0674	Milk	*K. pneumoniae*	42	404,130	56.64	85	5.61	–
Eg-BVM8	17Y0676	Milk	*K. pneumoniae*	34	363,930	57.34	73	5.36	Novel
Eg-BVM9	17Y0678	Milk	*K. pneumoniae*	51	362,204	56.65	48	5.61	–
Eg-BVM10	17Y0680	Milk	*K. pneumoniae*	116	217,146	57.05	78	5.55	37
Eg-BVM11	17Y0682	Milk	*K. pneumoniae*	109	217,177	57.05	75	5.54	37

### 3.3 Multilocus sequence typing (MLST)

Multilocus sequence typing analysis demonstrated heterogeneity among the isolates. Eight isolates were assigned to four distinct sequence types (STs). ST37 was identified in Eg-BVM4 (catfish), Eg-BVM10 (milk), and Eg-BVM11 (milk). Three isolates were assigned to ST105, including Eg-BVM2, Eg-BVM3, and Eg-BVM5, all from catfish. Eg-BVM1 (yogurt) was assigned to ST515, while Eg-BVM6 (catfish) belonged to ST3063. An isolate from yogurt (Eg-BVM1) was assigned to ST515 and an isolate from catfish (Eg-BVM6) was assigned to ST3063 (Table 1). The isolate Eg-BVM8 (milk) carried a novel allelic profile that did not match any known ST, representing a potentially new lineage (novel ST). Eg-BVM7 and Eg-BVM9 (both from milk) had incomplete allelic profiles, preventing assignment to a recognized ST. These results highlighted the genomic heterogeneity of *K. pneumoniae* across different food sources.

### 3.4 SNP based phylogenetic analysis

**FIGURE 1:**
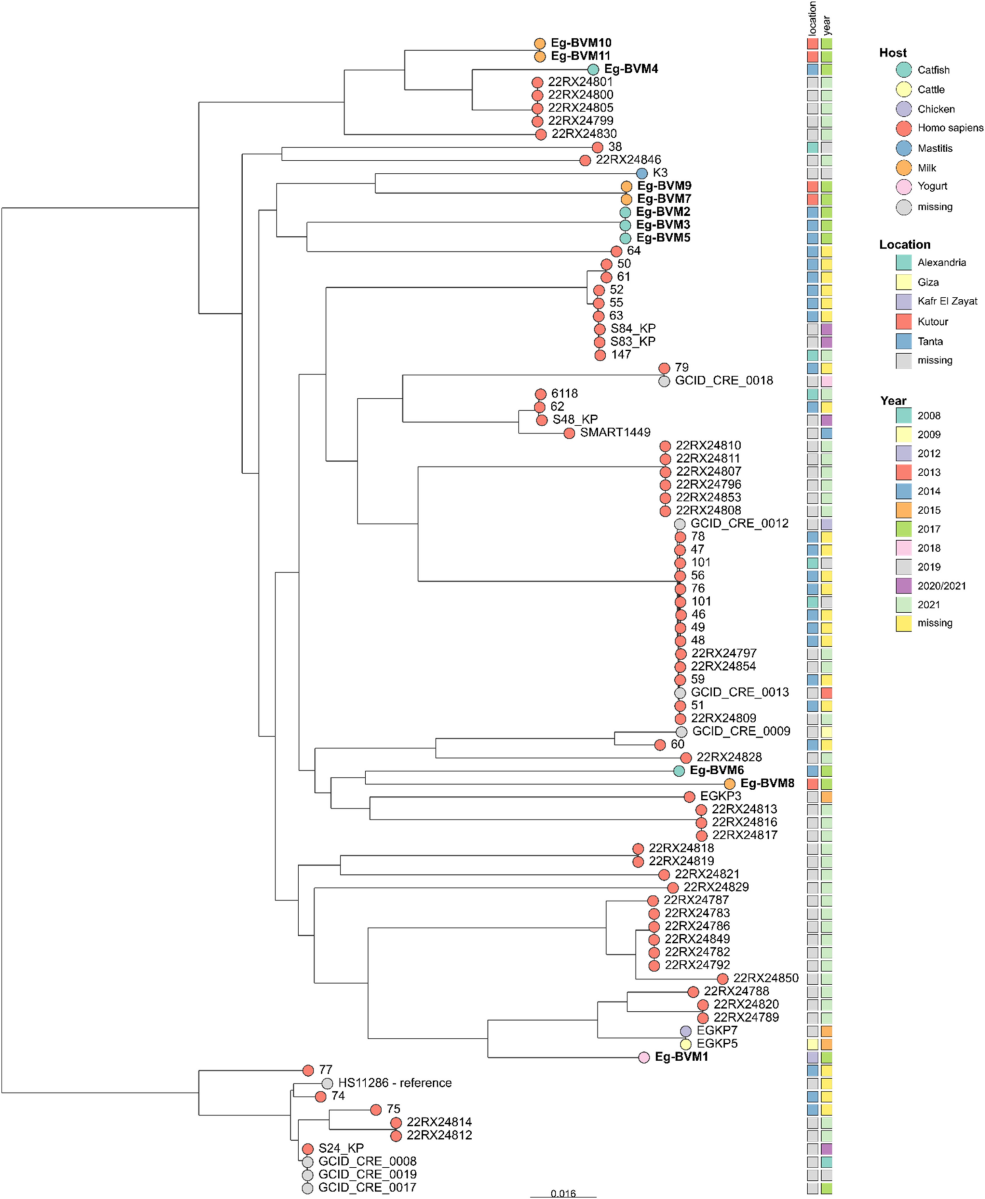
Maximum likelihood tree based on cgSNP analysis of *K. pneumoniae* isolates with source, location and year of isolation. The tree was rooted to the clade comprising the reference genome HS11286. The bar indicated substitutions per site.

Pairwise SNP distance analysis and phylogenetic reconstruction revealed both clonal clustering and substantial genetic divergence among the *K. pneumoniae* isolates. Within our food derived isolates, three groups were evident. The first comprised Eg-BVM2 (catfish), Eg-BVM3 (catfish), and Eg-BVM5 (catfish), which differed by only 1–5 SNPs. The second cluster included Eg-BVM7 (milk) and Eg-BVM9 (milk), which were separated by only 6 SNPs. The third cluster included Eg-BVM10 and Eg-BVM11 (milk) which differed by only 4 SNPs. Those groups were positioned in close proximity to Egyptian clinical isolates in the phylogenetic tree, suggesting potential epidemiological links between foodborne and human strains (Figure 1).

In contrast, several isolates formed divergent lineages. For example, Eg-BVM6 (catfish) was separated by more than 19,000 SNPs from other isolates. Eg-BVM10 and Eg-BVM11 (milk) also emerged as singletons, differing from Eg-BVM4 (catfish) by ∼10,800 SNPs. These isolates did not cluster with clinical isolates, highlighting the presence of genetically distinct *K. pneumoniae* lineages within food products.

The yogurt isolate Eg-BVM1 was genetically distinct, separated by ∼18,500–19,000 SNPs from the closest catfish or milk isolates. In the phylogenetic tree, it appeared as an independent lineage, not clustering with either foodborne strains or Egyptian clinical isolates (Figure 1). This pattern indicates that Eg-BVM1 represents a divergent lineage relative to the other sampled isolates and underscores the genomic diversity of *K. pneumoniae* recovered from different food products.

Comparison with 77 foreign (not belonging to this study) Egyptian clinical isolates revealed that the foodborne isolates (Eg-BVM2/3/5 and Eg-BVM7/9) are in the same branch with human clinical strains, underlining their potential clinical relevance. Conversely, the divergent isolates (Eg-BVM6, Eg-BVM8, and Eg-BVM10) remained genetically distant, reflecting the coexistence of unique food associated *K. pneumoniae* lineages.

### 3.5 *In silico* determination of resistance determinants and virulence associated genes

Whole genome sequencing confirmed the presence of a broad repertoire of resistance associated genes across all isolates (Supplementary Table 1). All isolates harbored the *oqxA* and *oqxB* efflux pump genes, which are associated with decreased susceptibility to quinolones and phenicols. In addition, all isolates carried multiple multidrug efflux pump determinants including *acrA*, *acrB*, *acrD*, *emrR*, *marA*, *mdtB*, *mdtC*, *kpnE*, *kpnF*, *kpnG*, and *kpnH*, supporting a broad intrinsic resistance profile. Among the β-lactamase genes identified, the *bla*SHV family was universally present across all isolates, with several allelic variants detected (*bla*SHV-1, *bla*SHV-11, *bla*SHV-110, *bla*SHV-145, *bla*SHV-187, and *bla*SHV-65). All isolates remained phenotypically susceptible to carbapenems (imipenem, meropenem, ertapenem) and no carbapenemase genes were detected.

Various *fosA* alleles (*fosA5*, *fosA6*, *fosA3*) were present, but their presence did not correlate with resistance, as all isolates were susceptible to fosfomycin. Efflux pump genes of the *acr*AB-*tol*C system (*acrA*, *acrB*, *acrD*), along with *oqxAB* variants and regulators (*marA*, *ramA*, *emrR*), were detected in all isolates; however, these determinants did not manifest phenotypically, as the isolates remained susceptible to fluoroquinolones, tetracyclines, and phenicols.

Metal-associated gene clusters, including silver (*silABCEFGPRS*), copper (*pcoABCDERS*), and arsenic (*arsABDR*) resistance operons, were widely distributed among the isolates. These determinants are typically linked to metal tolerance and may support environmental persistence or adaptation to food-processing environments.

Virulence-associated determinants were also detected, including siderophore systems such as yersiniabactin (*ybtA*, *ybtE*, *ybtP–ybtX*) and enterobactin-related genes (*entAB*, *fepC*, *fepG*, *fyuA*, *irp1*, *irp2*). In addition, components of the type I fimbrial cluster (*ecpABCDE*) were identified in most isolates, indicating the presence of virulence-associated features that may support environmental survival and host interaction.

## 4 Discussion

*Klebsiella pneumoniae* remains one of the most significant AMR pathogens worldwide and is listed by the World Health Organization (WHO) as a critical priority 1 pathogen in 2024 due to its ability to acquire carbapenemases, extended-spectrum β-lactamases (ESBLs), and colistin resistance determinants. This bacterium is responsible for severe community and hospital infections, including bloodstream infections, ventilator associated pneumonia, urinary tract infections, and neonatal sepsis (Lewis et al., 2022). Global genomic analyses have highlighted the rapid spread of epidemic high risk clones such as ST258, ST11, ST147, and ST307 alongside hypervirulent lineages carrying *rmpA*, *iroBCDN*, or *iucABCD* loci (Holt et al., 2015; Martin et al., 2023). The inclusion of *K. pneumoniae* in the WHO priority pathogen list highlights the importance of characterizing not only clinical reservoirs but also non-clinical sources, including food, which may act as underrecognized vehicle for human exposure.

The detection of *K. pneumoniae* in milk and yogurt samples in this study is consistent with previous reports identifying dairy herds, particularly cows affected by mastitis as important reservoirs of this pathogen, with recovery of *Klebsiella* spp. from raw milk reported globally (Song et al., 2023). Contamination may arise from infected udders, inadequate hygiene during milking or processing, or environmental exposure within farms. On the other hand, isolates of *K. pneumoniae* from catfish are concordant with studies showing that the bacterium inhabits aquatic ecosystems including fish ponds and surface waters and can colonize freshwater fish for human consumption (Cerdeira et al., 2020; Barati et al., 2016). These findings suggest that both animal associated and environmental reservoirs contribute to the circulation of *K. pneumoniae* in food chains, emphasizing the need for comprehensive surveillance of dairy and aquaculture products.

There is growing concern that foodborne *K. pneumoniae* may represent a source of human colonization and subsequent infection. Recent genomic studies have documented clinically relevant lineages and AMR determinants in food products and food related environments. In Germany, Klaper et al. (2021) performed a genome based analysis of *Klebsiella* isolates from animals and food products and identified MDR *K. pneumoniae* carrying ESBL genes and virulence associated loci in several food matrices. A European multicentric study by Rodrigues et al. (2022) further showed a high prevalence of *K. pneumoniae* species complex isolates in retail food, with some food isolates sharing genotypes with human clinical strains, reinforcing the view of food as a potential vehicle of colonization. Reviews within the One Health framework similarly emphasize the detection of hypervirulent and AMR *K. pneumoniae* in food matrices and the associated public health hazard (Hu et al., 2021; Cortés-Sánchez et al., 2025). Foodborne exposure may facilitate intestinal colonization, which is a recognized risk factor for invasive infection and for horizontal gene transfer of resistance determinants within the human intestinal microbiota. Even when resistance genes remain phenotypically silent in food isolates, their presence on mobile genetic elements is concerning, as plasmid mediated determinants can disseminate rapidly under antibiotic selective pressure (Hu et al., 2021; Thorpe et al., 2022).

A key finding of the present study is the predominantly susceptible antimicrobial phenotype observed among food derived *K. pneumoniae* isolates, with resistance largely limited to piperacillin in a subset of strains. Comparable susceptibility patterns have been reported in other investigations of foodborne *K. pneumoniae*. In Germany, a study demonstrated that animal and food associated isolates generally exhibited lower resistance levels than clinical strains, despite harboring intrinsic β-lactamase genes (Klaper et al., 2021). A similar study from Egypt reported that Enterobacterales recovered from Egyptian farms frequently carried ESBL and carbapenemase genes but did not consistently express phenotypic resistance, underscoring the gap between genomic potential and antimicrobial susceptibility (Elshafiee et al., 2022). Although these isolates remain susceptible to most tested antibiotics, they possess a diverse array of AMR genes that confer resistance to multiple antimicrobial classes, including ß-lactams, aminoglycosides, tetracyclines, cephalosporins, phenicols, quinolones, and heavy metals. *Klebsiella* species are commonly detected in environmental sources such as surface water, soil, sewage, plants, and wastewater, which function as reservoirs external to the human host. These bacteria can acquire AMR genes from the environment and subsequently transfer them to humans, primarily through horizontal gene transfer and interactions between environmental and human-associated bacteria (Alhaji et al., 2025; Akinyemi and Abdelmegid, 2025). Selection pressures outside clinical settings, including disinfectants, heavy metals in wastewater, agricultural runoff, and aquaculture, eliminate susceptible bacteria and promote the proliferation of resistant strains. *Klebsiella* strains harboring AMR genes gain a survival advantage, resulting in increased prevalence within these environments. The combined effects of environmental reservoirs, horizontal gene transfer, and antibiotic pollution facilitate the emergence of resistant *Klebsiella*, which may enter human populations via water, food, and surfaces, and are further propagated in healthcare environments (Wyres and Holt, 2018; Pedersen et al., 2020). Consistent with our findings, genomic analysis of *K. pneumoniae* isolates from wastewater treatment plant (influent and effluent) identified multiple *bla*SHV variants and *fos*A genes in environmental samples (Mabeo et al., 2025). This demonstrates that both types of AMR genes persist in environmental *Klebsiella*.

Several factors may explain the low resistance observed in this study. First, food production and aquaculture environments typically exert less antibiotic selective pressure than clinical settings, reducing the likelihood of multidrug resistant clone expansion. This principle is well documented in genomic epidemiological studies of *K. pneumoniae*, highlighting the strong association between antimicrobial exposure and the emergence of high risk, hospital adapted lineages (Lam et al., 2018). Second, although *bla*SHV genes (*bla*SHV-1, -10, -11, -110, -65 and -187) were common, the specific alleles detected are not produced an extended-spectrum phenotype, so genetic presence alone does not imply cephalosporin resistance (Bradford, 2001; Bush and Jacoby, 2010). Third, some resistance determinants identified by WGS may be transcriptionally silent or expressed at low levels, as antimicrobial resistance phenotypes often depend on promoter strength, gene copy number, and regulatory activation. This lack of phenotypic expression despite the genetic presence of AMR determinants has been documented in Enterobacterales, particularly in isolates from environmental or low-selective-pressure settings (Wyres and Holt, 2018; Holmes et al., 2016). Finally, environmental and animal associated *K. pneumoniae* often prioritize traits related to ecological persistence, such as nutrient scavenging, biofilm formation, or metal tolerance rather than maintaining energetically costly multidrug resistance mechanisms (Podschun and Ullmann, 1998; Wyres and Holt, 2016).

In this study, genotype–phenotype correlation was limited. Although all isolates carried multiple *bla*SHV and *fosA* variants, no cephalosporin or fosfomycin resistance was observed, consistent with the fact that many SHV alleles are narrow spectrum enzymes with limited activity against extended spectrum cephalosporins and carbapenems (Bradford, 2001; Bush and Jacoby, 2010). The only instance of partial correlation was piperacillin resistance, observed in isolates carrying SHV variants capable of penicillin hydrolysis, which is in line with the greater susceptibility to penicillins than cephalosporins at low level SHV expression. The variability in piperacillin susceptibility despite the universal presence of *bla*SHV alleles suggests that differences in allelic type, promoter strength, regulatory factors, or gene expression may influence the degree of β lactam hydrolysis. Thus, piperacillin resistance represents the clearest example in this dataset where genotypic determinants partially align with phenotypic non-susceptibility, reflecting the functional diversity of the SHV β-lactamase repertoire. Efflux pump systems (*acr*AB–*tol*C, *oqx*AB) and their regulators (*ramA*, *marA*, *emrR*) were present genomically but did not manifest as clinically relevant resistance, reflecting likely basal expression in these food derived isolates. No genotype–phenotype associations were observed for colistin or carbapenems, consistent with the absence of *mcr* genes, carbapenemase encoding genes, and the uniformly carbapenem susceptible phenotypes. All isolates remained phenotypically susceptible to carbapenems (imipenem, meropenem, ertapenem) and no carbapenemase genes were detected. Mutations were observed in genes associated with outer membrane permeability (e.g., porin-related *omp* loci) and LPS regulatory pathways (*pmrB*). However, reduced carbapenem susceptibility in *K. pneumoniae* is most commonly associated with carbapenemase production or with decreased permeability (porin loss/modification) in combination with ESBL or AmpC β lactamases; porin changes alone are often insufficient to confer clinically significant carbapenem resistance (Kaczmarek et al., 2006; Martínez-Martínez, 2008). Consistent with this, *pmrB* alterations are primarily described in the context of polymyxin (colistin) resistance rather than carbapenem resistance (Cannatelli et al., 2014). Overall, these observations reinforce that the presence of resistance genes alone does not reliably predict phenotypic resistance, a phenomenon widely documented for environmental and foodborne *K. pneumoniae* strains (Klaper et al., 2021; Wyres and Holt, 2016).

Multilocus sequence typing analysis identified clinically relevant lineages among food derived *K. pneumoniae* isolates. ST105 (Eg-BVM2, Eg-BVM3, Eg-BVM5; catfish) and ST37 (Eg-BVM4 from catfish; Eg-BVM10 and Eg-BVM11 from milk). ST37 has been reported as one of the most frequent sequence types among *K. pneumoniae* isolates obtained from human patients in Egypt (Soliman et al., 2024), indicating shared high risk clones circulating between food and clinical settings. Eg-BVM1 (yogurt, ST515) and Eg-BVM6 (catfish, ST3063) belonged to less common STs, while Eg-BVM8 (milk) represented a novel ST. For example, BVM7 and BVM9 (milk) had incomplete allelic profiles due to presence of one novel alles in each, which prevented ST assignment, but SNP clustering placed them near clinical isolates. Together, these findings highlight both the presence of high risk clinical STs in food products and the emergence of novel or divergent lineages within the Egyptian food chain (Table 2).

**TABLE 2:** Multilocus sequence typing (MLST) of *K. pneumoniae* isolates with sources and clinical relevance.

Sequence type (ST)	Isolates (Eg-BVM/Kp)	Source(s)	Clinical link (Egyptian SRA)	Notes
ST105	Eg-BVM2, Eg-BVM3, Eg-BVM5	Catfish	Yes – detected in multiple Egyptian clinical isolates	High-risk clone; food–clinical overlap
ST37	Eg-BVM4, Eg-BVM10, Eg-BVM11	Catfish, milk	Yes – reported in Egyptian clinical isolates	International high-risk clone, MDR-associated
ST515	Eg-BVM1	Yogurt	Rarely clinical, reported sporadically	Distinct yogurt lineage
ST3063	Eg-BVM6	Catfish	No Egyptian clinical match	Divergent lineage, geographically restricted
Novel ST	Eg-BVM8	Milk	No clinical match	Potentially new lineage
Unassigned	Eg-BVM7, Eg-BVM9	Milk	SNP clustering near clinical isolates	Incomplete allelic profiles

The detection of clinically relevant sequence types (ST37, ST105) and of a novel ST in this study underscores the importance of expanding genomic surveillance within Egyptian food systems. WGS enables high resolution tracking of clonal lineages, early detection of emerging high risk clones, and identification of hidden reservoirs of resistance or virulence determinants. Further on, more geographically diverse sampling is needed to fully understand the transmission dynamics between food, animals, the environment, and clinical populations. The integration of WGS into routine food safety monitoring is increasingly recognized as an essential component of global AMR surveillance frameworks (Koutsoumanis et al., 2019). Internationally, WGS has been increasingly applied to characterize *K. pneumoniae* in food and animal reservoirs, revealing patterns that partially align with the findings of the present study. WGS based investigations from Europe and East Asia have demonstrated that foodborne *K. pneumoniae* can include lineages that overlap with those detected in human clinical settings, alongside with more divergent, environmentally adapted strains (Klaper et al., 2021; Liao et al., 2020). These findings indicate that retail food products may harbor heterogeneous *K. pneumoniae* populations with varying public health relevance, although genomic data alone cannot establish transmission directionality.

In contrast, the interest of authorities and researchers from Gulf countries remains heavily weighted toward clinical surveillance. Multiple WGS studies from Saudi Arabia, the United Arab Emirates, Qatar, and neighboring states have documented the circulation of carbapenem resistant *K. pneumoniae* and high risk epidemic clones in hospital settings, often associated with OXA-48-like or NDM carbapenemases and extensive antimicrobial resistance profiles (Zowawi et al., 2013; Thomsen et al., 2023; Tsui et al., 2023). Thus, comparable genomic investigations targeting food, animal, or environmental reservoirs in the Gulf region are scarce. As a result, the potential contribution of upstream reservoirs particularly food products to the broader epidemiology of the pathogen in human medicine remains insufficiently characterized. This imbalance highlights a clear One Health gap, as hospital based genomic data are not routinely complemented by systematic WGS surveillance of food systems.

Within Egypt and the wider North African region, several studies have documented the presence of *K. pneumoniae* and other Enterobacterales in food products, fresh products of farms, irrigation water, and seafood, in some cases ESBL or carbapenemase associated genes were reported (Alhaji et al., 2025; Mohammed et al., 2024; Elshafiee et al., 2022; Akinyemi and Abdelmegid, 2025). However, many of these investigations rely on phenotypic susceptibility testing and targeted gene detection using PCR rather than comprehensive genomic analysis. The present study extends the regional knowledge by applying WGS to ready-to-eat food products obtained from supermarkets and by contextualizing foodborne isolates within a phylogenetic framework that includes Egyptian “clinical” genomes. Notably, while some food derived isolates clustered with clinical strains, others formed highly divergent lineages, underscoring the coexistence of potentially human associated and environmentally adapted *K. pneumoniae* populations within the food chain.

## 5 Conclusion

This study provides a whole genome based characterization of *K. pneumoniae* isolated from ready-to-eat food products in Egypt and highlights the presence of both clinically relevant and genetically distinct lineages within the food chain. Although most isolates exhibited a predominantly susceptible antimicrobial phenotype, they harbored a diverse repertoire of resistance associated and virulence related genes, underscoring the distinction between genomic potential and phenotypic expression of antimicrobial resistance. The detection of sequence types previously associated with human infections, along with a novel lineage, suggests that food products may serve as reservoirs for heterogeneous *K. pneumoniae* populations with varying public health relevance. These findings emphasize the importance of integrating phenotypic susceptibility testing and whole genome sequencing in surveillance efforts and demonstrate the need for expanded One Health surveillance of food systems to better understand the ecology and transmission dynamics of *K. pneumoniae* in Egypt and beyond.

## Data Availability

All data generated in this study are provided within the article and its Supplementary materials. Raw sequencing data produced in the current study have been deposited in the European Nucleotide Archive (ENA), with a project accession number PRJEB106138.

## Footnotes

1
https://gitlab.com/FLI_Bioinfo/WGSBAC

2
https://github.com/tseemann/shovill

3
https://github.com/tseemann/abricate

4
https://github.com/tseemann/mlst

5
https://github.com/tseemann/snippy

## Supplementary material

Supplementary Table 1 Metadata of isolates, results of susceptibility testing, WGS analysis and assembly statistics including MLST, as well as AMR and virulence data.

Supplementary Table 2 The accession numbers of all *K. pneumoniae* isolates used for comparison in the current study.

Supplementary Table 3 Taxonomic classification of *K. pneumoniae* isolates with Kraken2.
